# Digitally supported interprofessional interaction in healthcare—a scoping review

**DOI:** 10.3389/fdgth.2025.1688989

**Published:** 2025-12-12

**Authors:** Stefanie Sauter, Kim Nordmann, Michael Schaller, Marie-Christin Redlich, Florian Fischer

**Affiliations:** Bavarian Research Center for Digital Health and Social Care, Kempten University of Applied Sciences, Kempten, Germany

**Keywords:** digital healthcare, digital health technologies, healthcare professionals, implementation science, health communication, digital collaboration

## Abstract

**Background:**

The increasing complexity of patient care and workforce shortages in healthcare systems necessitate improved interprofessional interaction. Digital technologies offer promising solutions to facilitate such interaction across healthcare settings.

**Objectives:**

This scoping review aimed to identify, categorize, and assess digital technologies that support interprofessional interaction among healthcare professionals, using the NASSS framework to evaluate their implementation context and impact.

**Methodology:**

A systematic search was conducted across five databases. The eligible studies examined digital tools enabling interaction between different professional groups in healthcare. Data from 407 studies were extracted and coded using four NASSS domains (Condition, Technology, Value Proposition, and Adopter System). Thematic analysis and visualizations were employed to synthesize findings.

**Results:**

Seven primary technology categories were identified. Most technologies were implemented at the organizational level, primarily within hospital and intersectoral care settings, with oncology being the most common clinical focus. While many tools showed positive impacts on workflow efficiency, access to specialist expertise, and team communication, challenges relating to usability, data privacy, role ambiguity, and staff workload were also reported. Value propositions and impacts on staff varied significantly across technologies.

**Conclusion:**

Digitally supported interprofessional interaction holds promise for enhancing communication, collaboration, and efficiency in delivering healthcare. However, successful adoption depends on aligning technological design with clinical workflows, involving end-users in development, and addressing regulatory, ethical, and organizational challenges.

## Introduction

Demographic, social, and technological changes are key contributors to the growing strain on the healthcare system. These processes influence both the type and volume of healthcare services required. Simultaneously, there is an increasing mismatch between the number of healthcare workers and the rising demand, with projected shortages of nurses and physicians in the coming years (1–3). Against this background, digital technologies may transform interprofessional interaction between healthcare professionals, especially for complex conditions or chronic disease management. Interprofessional interaction in this context describes any interaction between healthcare professionals, including collaboration, communication, cooperation and coordination using digital tools, also described as D4C (4). Digital healthcare solutions—such as virtual multidisciplinary team meetings or shared electronic records—facilitate the exchange of knowledge between healthcare professionals and enhance efficiency and improve patient outcomes (5, 6).

Despite these benefits, several challenges regarding technical, infrastructural, organizational and financial barriers, for example, hinder the sustainable implementation and adoption of digital technologies for interprofessional interaction in the healthcare system (7–10). Factors such as power imbalances between healthcare professionals, unclear roles and responsibilities, and concerns about the impact of these technologies on professional identity contribute to the failure or non-adoption of digital tools in the context of interprofessional interaction (11). Achieving widespread usage and the additional benefits of these technologies depends on multiple factors that affect their short- and long-term implementation and scalability.

There are several frameworks designed to guide the systematic assessment of potential barriers and facilitators to implementation (12, 13); Implementing technology is complex, thus actionable frameworks are needed. One such framework is the comprehensive *Consolidated Framework for Implementation Research* (CFIR), which synthesizes constructs from a range of implementation theories and frameworks (13). However, the focus of the CFIR delivers a broader range of interventions and addresses complexity as one of many intervention characteristics. To better understand the complexities involved in implementing and adopting technology-supported innovations in healthcare and social care settings, Greenhalgh et al. (12) developed the NASSS framework (Nonadoption, Abandonment, Scale-up, Spread, and Sustainability). This framework highlights the complexities and uncertainties inherent in projects for implementing technology, considering long-term sustainability of innovations. Despite the importance of interprofessional interaction and the growing availability of digital communication tools, current studies often either focus on isolated aspects such as a single technology or a specific sector, or analyze the very broad context of “digital health technologies”. Therefore, the aim of this study was to conduct an extensive scoping review across all healthcare settings and professions, disease spectra, and technologies in order to elicit 1) the proposed value and the impact of digital support on interprofessional interaction and 2) the most commonly used technologies for interprofessional interaction within their respective adopter system.

## Methodology

The scoping review was conducted in accordance with the Joanna Briggs Institute methodology (14), with comprehensive methodological details and the full search strategy provided in the published protocol (15), and is reported in compliance with the PRISMA-ScR guidelines (16).

### Search strategy

The search string—including various keywords for interaction, healthcare settings, and digital tools—was tailored to five databases (MEDLINE, CINAHL, Embase, PsycInfo, and Scopus) in December 2022. Given the rapid advancements of technologies during the last decade, the search was limited to studies published in English, French, German, Portuguese, or Spanish from 2012 onward (15). KN additionally manually screened the reference lists of finally included records to elicit eligible articles not identified via the database search.

### Eligibility criteria

All types of primary research methods—including qualitative, quantitative, and mixed-method approaches—as well as their corresponding study designs were included. In addition, secondary research methods such as reviews, meta-analyses, and meta-syntheses were considered. Opinion articles reporting on any healthcare setting were also included. To be included, papers had to investigate digital tools facilitating interaction between (1) at least two distinct groups of healthcare professionals, or (2) healthcare professionals in similar roles working in different organizations. Conference abstracts, book chapters, sources without full-text access, and studies focusing primarily on interactions between healthcare providers and patients or in the context of healthcare education were excluded.

### Assessment and selection of records

**Figure 1:**
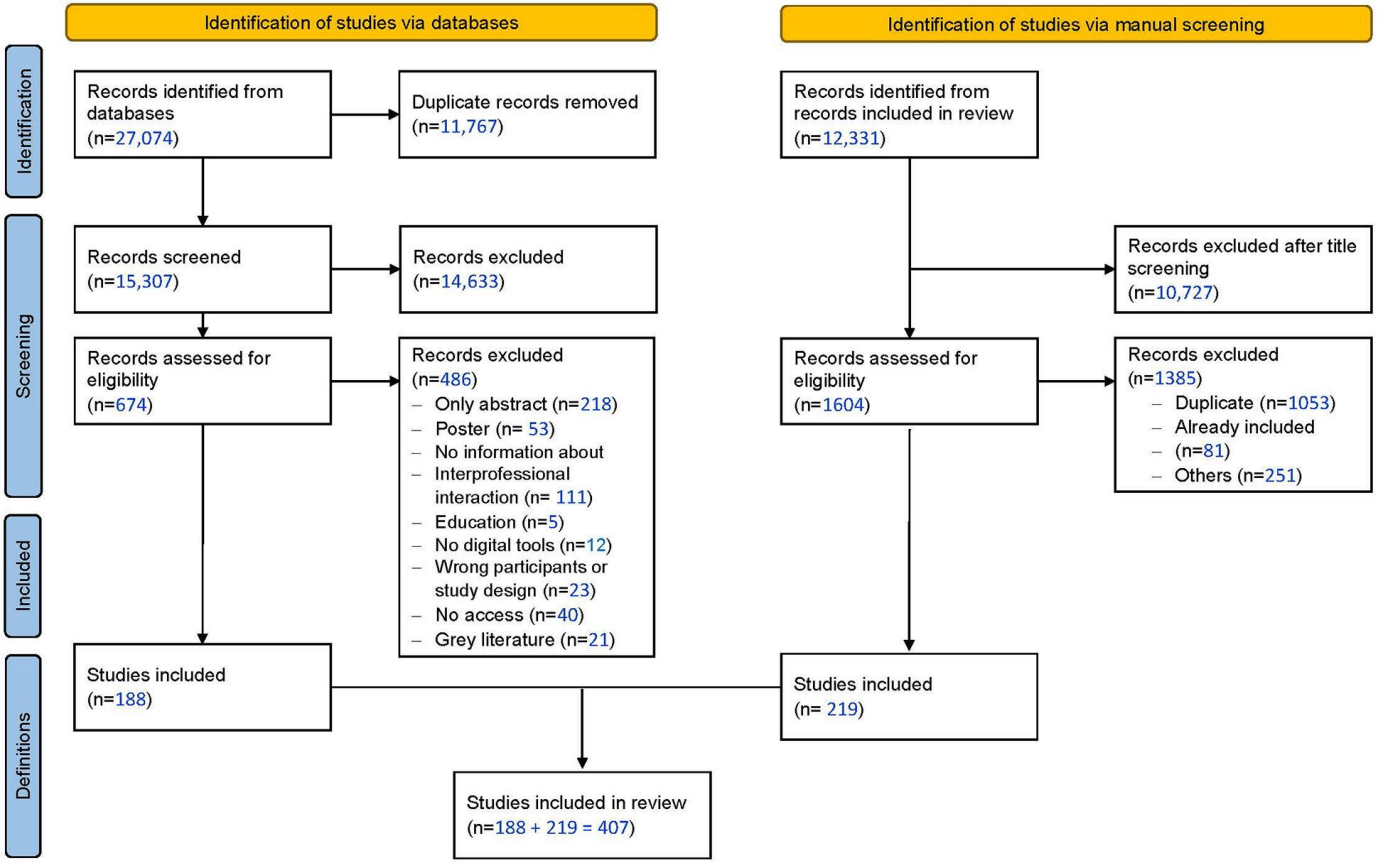
Prisma flow diagram for study selection and derivation of definitions [Adapted from Page et al. (18)].

All bibliographic data was imported to Covidence (Veritas Health Innovation Ltd, Melbourne, Australia). Overall, 27 074 records were retrieved from the databases. After identifying duplicates, 15 307 records remained. The first 50 abstracts were screened by all authors (KN, SS, MS, MCR, FF) and discrepancies discussed directly to ensure a consistent assessment. The remaining abstracts were screened by two researchers each out of the group of five researchers, with a third researcher resolving any conflicts. Following the screening of titles/abstracts, 674 records proceeded to full-text review, with 188 ultimately being included in the dataset. The weighted average of the two researchers’ Cohen *κ* at the abstract screening stage was 0.22 and for the full-text screening 0.53, indicating a fair interrater agreement for abstract screening and moderate agreement for full-text screening (17, 18). A manual search of 12 331 references within these 188 records identified 1 604 additional records for screening, resulting in 219 additional studies being included in the review (see Figure 1).

### Data extraction and analysis

**Figure 2:**
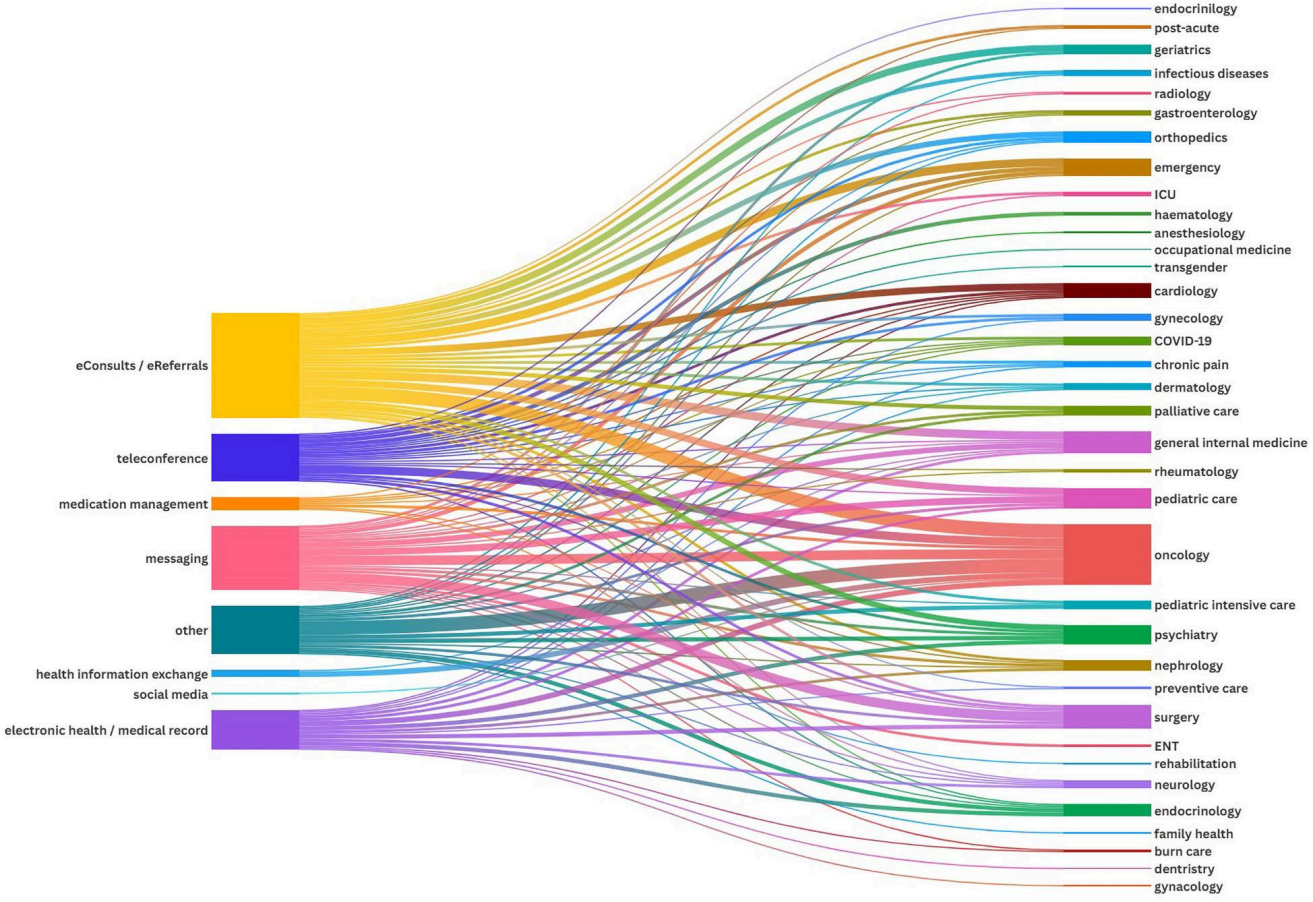
Visualization of the association between the different technologies and their use across different disease areas.

**Figure 3:**
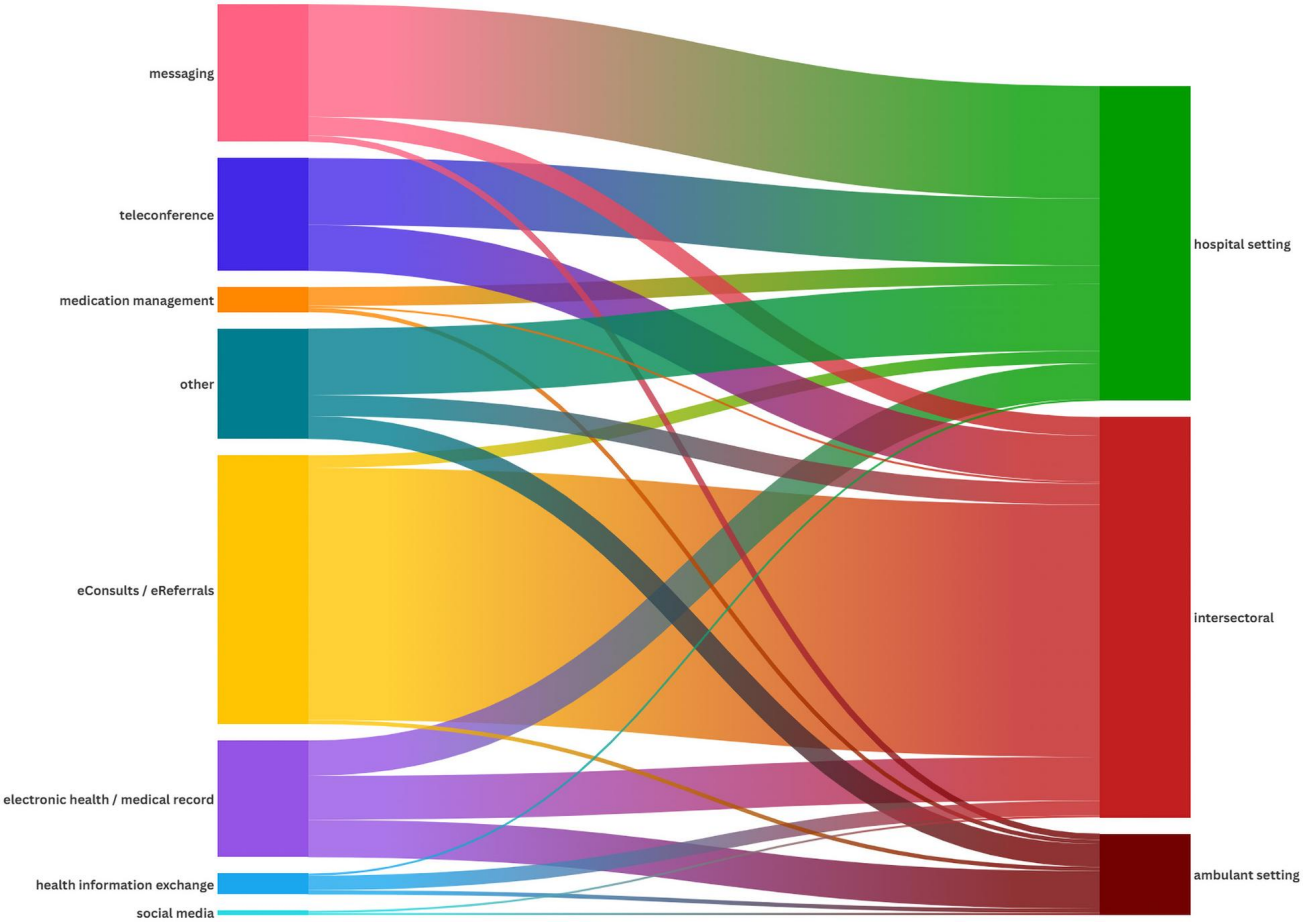
Visualization of the association between the different technologies and their use across different settings.

**Figure 4:**
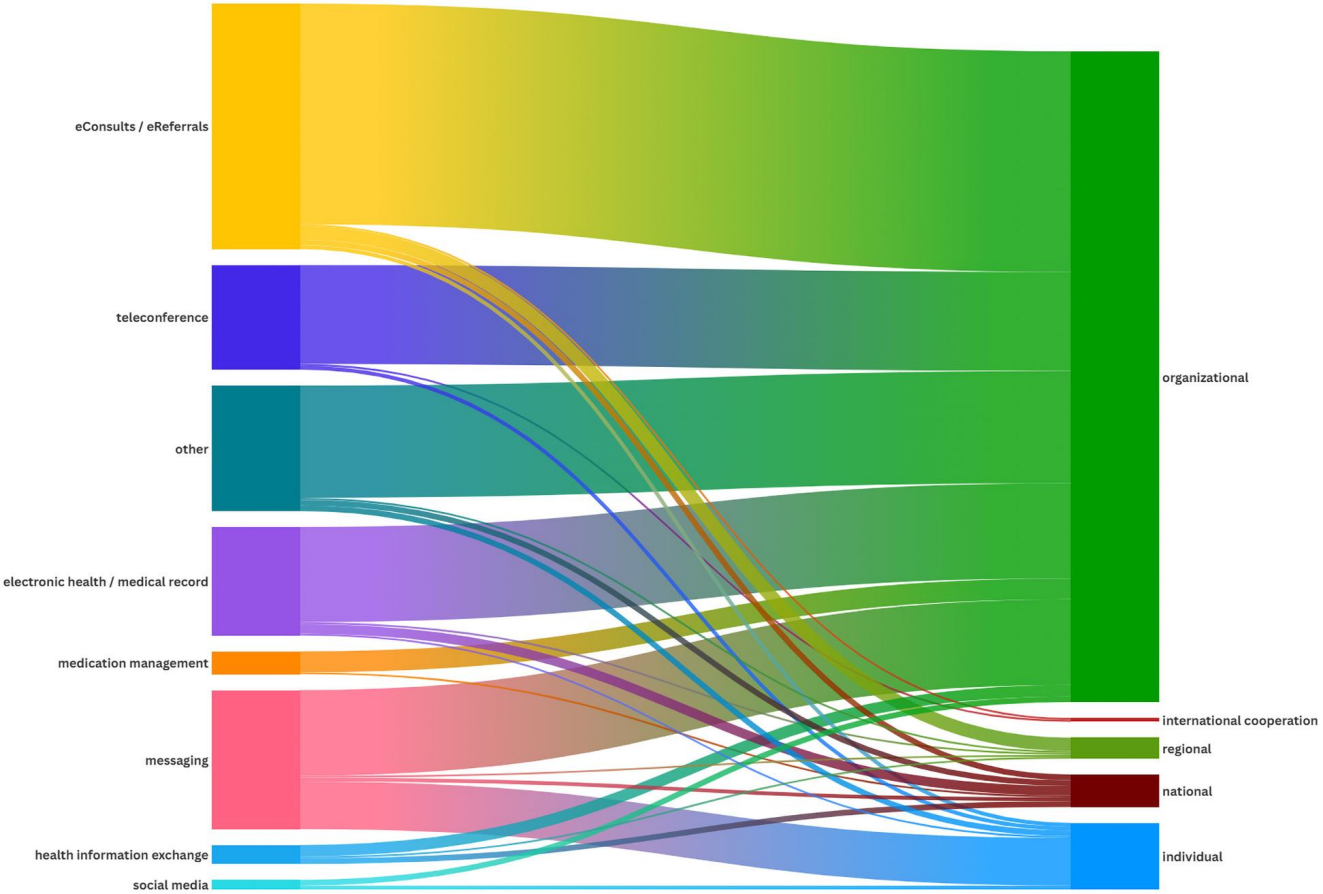
Visualization of the association between the different technologies and their levels of implementation.

**Figure 5:**
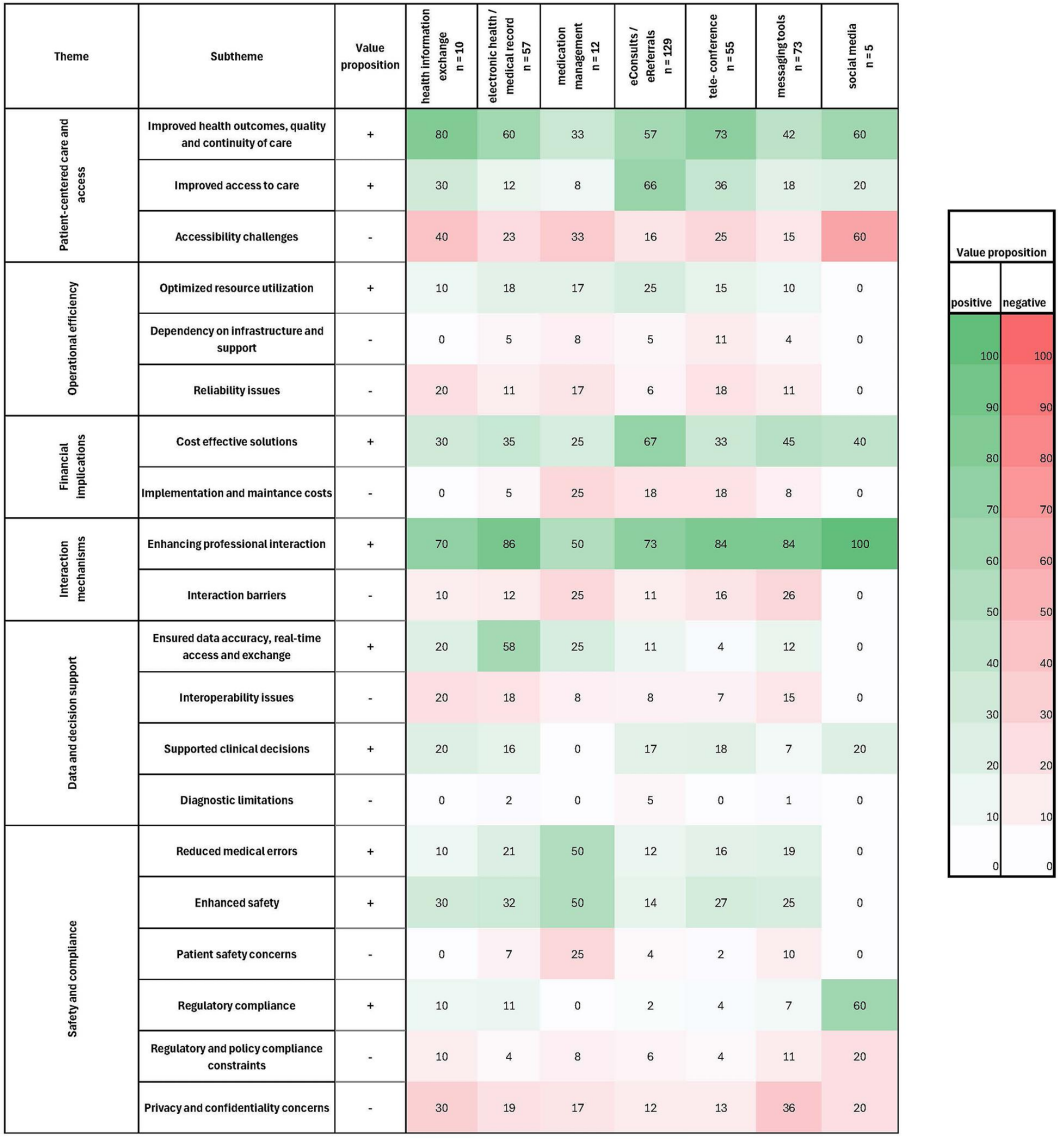
Heatmap of key technologies and their reported value proposition. Green colors indicate a positive value proposition, red colors a negative value proposition. The more intensive the color, the higher the percentage of articles on the respective technology that reported on this factor (+ and − indicating positive and negative effects on the adopter system, respectively).

**Figure 6:**
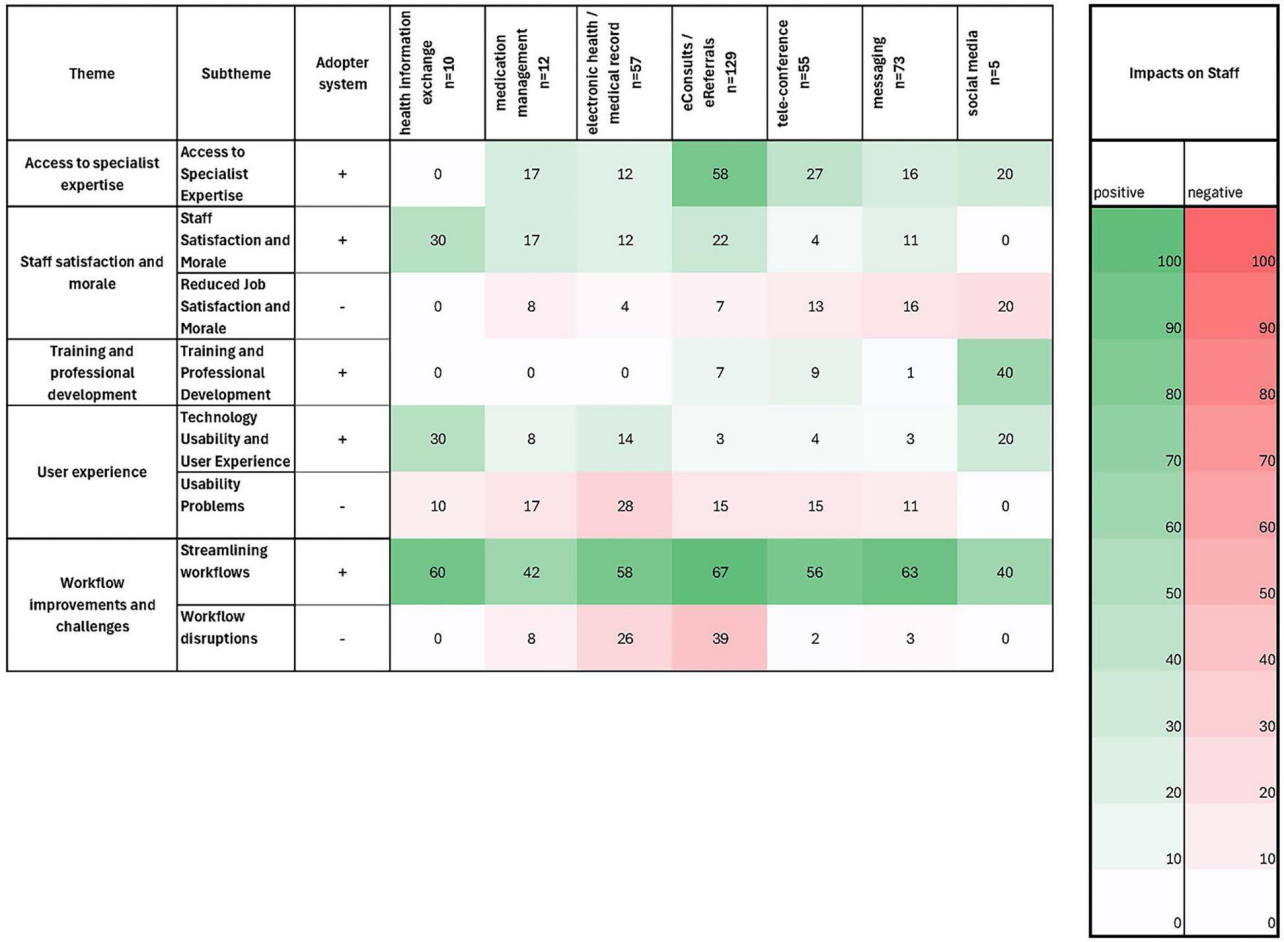
Heatmap of key technologies and their reported positive and negative impact on staff. The more intensive the color, the higher the percentage of articles on the respective technology that reported on this factor. (+ and − indicating positive and negative effects on the adopter system, respectively).

Overall, 407 records were imported into MAXQDA (Version 2022; VERBI GmbH, Berlin, Germany) and relevant sections coded based on the seven domains outlined in the NASSS framework [the Condition or Illness; the Technology; the Value Proposition; the Adopter System; the Organization(s); the wider System; Embedding and Adaptation over time] (12). In our analysis, we focused on the domains 1–4 of the NASSS framework for structuring the results: Domain 1 (**Condition**) refers to the illness or health condition being targeted by the technology; domain 2 (**Technology**) addresses the features of the technology itself, including its functionality, usability, data handling, and reliability; domain 3 (**Value Proposition**) looks at the value offered to stakeholders, including clinical effectiveness, cost-effectiveness, and the business case; and domain 4 (**Adopters**) considers the intended users of the technology (e.g., patients, clinicians, caregivers). To visualize the relationships between the domains and to highlight the complex interactions, Sankey charts were created for the technology and the disease spectra (Figure 2), technology use per setting (Figure 3), and the level of technology implementation (Figure 4), using Flourish (Canva UK Operations Ltd). For further analysis and visualization, heatmaps presenting the key point for both the Value Proposition (Figure 5) and the Adopter System (Figure 6) were created using Excel (Microsoft Office 2024).

For the purpose of this review, we focused on the group of healthcare professionals as adopters. In the NASSS framework, each domain contains specific questions, which were adapted, rephrased, or omitted to align with the objectives of this study—questions regarding the technology (domain 2) encompassed for example: “What are the key features of the technology?” and “What knowledge and/or support is required to use the technology?” (12). All adapted questions per domain are presented in Table 1.

**Table 1:** Adapted questions in reference to the NASSS (nonadoption, abandonment, scale-up, spread, and sustainability) frame work.

Domain	Questions
Condition	Why and in which setting was the technology introduced? Is a specific disease area mentioned?
Technology	What are the key features of the technology? What is the current use and uptake? What is the level of implementation?
Value proposition	What is the technology's positive value proposition in terms of e.g., desirability, efficacy, safety, and cost effectiveness, amongst others, excluding beneficial effects on staff?
Adopter system	What is the technology's desirability for staff? (Including beneficial effects on staff?)

The adapted questions for each domain were applied to the contributions included in the review, producing more than 1,000 pages of coded text. In an ancillary study, we could show in a structured, randomized, and blinded review process that an AI-based chatbot's summaries of scientific text passages (in the form of answers to the specific questions within the seven domains of the NASSS framework) were at least comparable to answers given by humans (19). Therefore, we used ZenoChat on each text snippet across all codes to extract the answers pertaining to the questions of each domain. ZenoChat's answers were then analyzed using thematic analysis (20) to identify and analyzing patterns and themes within the data. Further information on the use of ZenoChat for the analysis of the coded text can be found in Nordmann et al. (19).

## Results

Overall, 407 articles were included in the analysis (Figure 1) and analyzed according to the NASSS framework.

### NASSS domains 1 and 2: condition and technology

The analysis identified seven primary categories of technologies supporting interprofessional interaction: eConsults/eReferrals (*n* = 129, 32%), messaging tools (*n* = 73, 18%), electronic health records (*n* = 57, 14%), teleconferences (*n* = 55, 14%), medication management systems (*n* = 12, 3%), health information exchange systems (*n* = 10, 2%), and social media tools (*n* = 5, 1%). Additionally, 66 papers (16%) reported various other technologies that were either not clearly defined or described and did not align with any of the identified categories. Most technologies were implemented at an organizational level (*n* = 342, 84%), followed by implementation on an individual level, i.e., at the healthcare professional level (*n* = 25, 6%), and on a national level (*n* = 17, 4%). Only two technologies were implemented on an international level (21, 22) (see Figure 4). As described by Bonnardot et al. (21), Médecins Sans Frontières (MSF) launched a telemedicine support for field workers. The other international implementation was a teleconference program between a Jordanian and a Canadian children's hospital (22). The key features of the seven main technologies and their implementation level are detailed in the following section. In-depth information about each of the included studies is available in the Supplementary Material S1. The analysis also identified disease settings where technologies were primarily used, with oncology being the most prominent indication area (see Figure 2). However, only 237 papers clearly defined an indication area in terms of the underlying disease spectra.

#### Econsults and eReferrals

Predominantly used in an intersectoral healthcare setting (94%), eConsults enable asynchronous remote consultations between primary care providers and specialists, whereas the primary goal of eReferrals is to refer a patient to a specialist for further assessment or treatment. eConsults and eReferrals can enhance care coordination, reduce delays, and improve continuity of care. These tools enable remote consultations, addressing gaps in care and providing real-time support for both patients and providers. Most implementations (90%) occurred at organizational level (see Figure 3), for example connecting primary care providers with specialists to expand the use of eConsults for Medicaid patients in the UK (23).

#### Messaging tools

Mainly implemented in a hospital setting (82%), these tools include secure text messaging and instant messenger platforms such as WhatsApp. They enable rapid communication among healthcare professionals, facilitating real-time exchange of clinical information. Leveraging smartphones and other devices, these tools support diverse data formats—text, images, and videos—enhancing team collaboration, care coordination, and operational efficiency. Implemented at both organizational (62%) and individual levels (34%), they streamline interaction between healthcare professionals (see Figures 2, 3).

#### Electronic health/medical records (EHRs)

Electronic health records (EHRs) or Electronic Medical records (EMRs) improve access to treatment data, reports, and services, thus enhancing time management, care coordination, and patient outcomes. These systems enable seamless sharing of medical information, which supports interprofessional interaction and integrated care. Key functionalities include structured data storage, retrieval, and tools for discharge processes and emergency management, which facilitate interprofessional communication and informed decision-making. The majority (88%) were implemented at an organizational level, with 9% at national level (see Figures 2, 3).

#### Teleconferences

Primarily hospital-based (59%) and intersectoral (40%), teleconference technologies facilitate effective communication and decision-making among healthcare providers using audio-visual tools and virtual platforms. They enable multidisciplinary team meetings for comprehensive care, particularly in complex cases such as cancer treatment. Teleconferences were mainly used for oncological discussions. They served knowledge transfer, improved access to healthcare, and supported documentation. Most (95%) were implemented organizationally (see Figures 2, 3).

#### Medication management systems

Mainly hospital-based (75%), these tools streamline workflows via dashboards and digital platforms as a means of enhancing communication, data sharing, and interprofessional interaction. These systems support computerized physician order entry with integrated support for clinical decisions to improve prescribing accuracy and reduce errors. By implementing electronic prescribing and real-time insights, these tools optimize medication workflows, improve pharmaceutical care, and ensure medication safety across healthcare settings. Nearly all implementations (92%) occurred at the organizational level (see Figures 3, 4).

#### Health information exchange (HIE) systems

Largely intersectoral (70%), HIE systems enable health data to be shared securely among healthcare providers, thus improving clinical decision-making and care outcomes. By integrating healthcare information, HIE systems support patient management, facilitate smooth transfers between care settings, and enhance communication, particularly in emergency departments. These systems provide critical summaries and actionable insights, thus improving coordination and efficiency in delivering care. Most of these systems (60%) were implemented at organizational level (see Figures 2, 3).

#### Social media tools

Used in ambulatory (50%) and intersectoral (50%) settings (see Figure 2), social media platforms—i.e., virtual communities with user-generated content—facilitate sharing clinical information and knowledge, and improve collaboration via these tools. They connect healthcare professionals to resources, and promote the dissemination of healthcare information while fostering community engagement in web-based environments. Social media tools were used at both organizational and individual level.

### NASSS domain 3: value proposition

Thematic analysis identified six overarching themes where the technology demonstrated both positive and negative value propositions: patient-centered care and access, operational efficiency, financial implications, interaction mechanisms, data and decision support, and safety and compliance. Figure 5 shows a heatmap displaying the accumulated value proposition per technology. By visually inspecting the heatmaps, it can be seen that eConsults and EMR/ HER are the most positively rated technologies, together with HIE. Medication management and messaging systems are the most negatively rated systems, respectively.

#### Patient-centred care and access

Digital technologies contributed to **improved health outcomes, quality and continuity of care** through timely care provision and seamless transitions of care enhancing handover communication and supporting shared care plans. Technologies such as eConsults further improved patient-centred care by **enhancing access to care** for hard-to-reach patients, and reducing travel and waiting times. However, some **barriers to accessibility** were identified, which were highest with regard to social media. These include the impersonal nature of digital interactions, which can deter adoption, and non-inclusive designs that limit usability for certain segments of the population.

#### Operational efficiency

Digital tools were seen to **optimize the utilization of resources by** minimizing unnecessary procedures, tests, or consultations, while improving the allocation of clinicians’ time and other resources. However, the success of these tools is contingent on a **stable infrastructure** with **ongoing support and maintenance**. **Reliability issues**, such as crashes and downtime, were reported for some tools, leading to workflow disruptions, provider frustration, and reduced operational efficiency.

#### Financial implications

Efficiency gains achieved by streamlining operations were reported across technologies, thus positioning digital tools as **cost-effective solutions**. Especially eConsults are reported to be cost-effective by reducing unnecessary in-person visits and making it possible to reach patients in under-served and remote areas, where implementing non-digital programs would have been expensive. Nevertheless, significant **negative cost implications** were also identified. Many technologies require significant upfront investment in infrastructure, tools, training and ongoing maintenance, updates and licensing costs. These implementation and maintenance costs were highest in medication management systems. A lack of generalized financing structures can result in out-of-pocket expenses for providers or place a disproportionate financial burden on smaller facilities.

#### Interaction mechanisms

Digital tools **enhanced professional interaction**, improved communication clarity and speed, and team collaboration. Notably, the technology was reported to flatten hierarchies within (multidisciplinary/multiprofessional) teams, aligning team members through equal access to information. However, **barriers** to effective interaction persisted or were exacerbated when tools were misaligned with the needs of healthcare professionals. In extreme cases, communication failures via digital tools can lead to misunderstandings and errors in patient care. Furthermore, the digital nature of interactions may limit the development of personal relationships among healthcare providers, thus potentially hindering effective team relations and dynamics.

#### Data and decision support

Digital tools **improved data accuracy** compared to paper-based systems by aggregating data from various sources and enabling **real-time access** for updating patient parameters. These systems also support clinical decision-making through the analysis of patient information. However, **interoperability issues** persisted, reducing data reliability and complicating decision-making processes.

#### Safety and compliance

Digital tools, such as medication management tools, enhanced **patient safety** by standardizing workflows and providing alerts for potential treatment risks, thereby reducing **medical errors** and ensuring **alignment with safety protocols** and evidence-based practices. Nonetheless, **safety concerns** arise if tools are not appropriate for the use case or users fail to adequately interact with the tools. Compliance with legal and regulatory standards remains inconsistent; some tools meet requirements, while others, such as WhatsApp, fail to ensure adequate data protection and confidentiality. This can compromise **patient privacy**, leading to mistrust among patients and anxiety for healthcare workers, who may fear legal or professional repercussions for inadvertent exposure of sensitive information. These concerns were present across all technologies, but highest in HIE and messaging tools. Additionally, tools to support clinical decision-making can occasionally provide **inaccurate diagnostic** results, thus potentially compromising patient safety and/or undermining trust in digital technologies.

### NASSS domain 4: adopter system (healthcare professionals)

The integration of new tools, technologies, and collaborative practices often redefines staff roles and professional identities within the healthcare environment. Workflow modifications frequently lead to the redefinition of roles, which requires staff to adapt their responsibilities to align with updated processes and navigate new boundaries in interprofessional relationships. Thematic analysis identified five overarching themes: access to specialist expertise, staff satisfaction and morale, training and professional development, user experience, and workflow improvement and challenges. Figure 6 shows a heatmap displaying the accumulated impact of technologies on the adopter system. Visually inspecting the heatmap reveals eConsults and social media to be the most positively rated technologies, together with HIE. However, eConsults are also the most negatively rated technology at the same time, as is EMR.

#### Workflow improvement and challenges

Digital tools streamline workflows by automating routine tasks and improving communication via secure messaging, smartphone apps, and centralized dashboards. By standardizing processes such as scheduling, prescription processing, and documentation, they reduce cognitive load, minimize errors, and allow staff to focus more on patient care. Innovations such as eConsults streamline specialist access, and optimize resource allocation. However, technological systems can redistribute responsibilities, thus increasing workloads and creating role ambiguity. Uneven adoption of technology among team members exacerbates confusion and disrupts cohesion. Challenges such as frequent digital alerts and multitasking demands can distract, reduce focus, and exacerbate cognitive strain. Impersonal electronic communication diminishes trust and collaboration, while administrative burdens and fragmented workflows contribute to dissatisfaction, burnout, and errors in care delivery.

#### Access to specialist expertise

Digital tools bridge geographic and logistical gaps, thus improving primary care providers’ access to remote specialists. Asynchronous tools such as eConsults reduce delays and unnecessary referrals, while videoconferencing enhances interprofessional coordination, which improves planning of treatments and the efficiency of care. Access to specialized knowledge, such as oncology or mental health expertise, empowers primary care providers to manage complex cases confidently. Furthermore, access to centralized digital repositories, decision-support tools, and platforms equips professionals with up-to-date guidelines, databases, and research. Collaborative platforms, including secure forums and virtual team meetings, promote exchange of knowledge, the discussion of challenging cases, and a culture of continuous learning and support.

#### Training and professional development

Digital tools such as eConsults integrate case-based learning into continuing medical education, thus promoting professional growth. These platforms provide advanced educational materials, peer-to-peer learning and real-time feedback, which can boost confidence and expertise. For providers in remote or under-served areas, these technologies improve access to training, thereby enhancing job satisfaction and retention. By reinforcing clinical decision-making and offering real-life case management experiences, they reassure practitioners and foster skill enhancement.

#### Staff satisfaction and morale

Streamlining workflows using digital technologies can reduce burdensome tasks, improve efficiency, and enhance staff satisfaction. Technologies such as telemedicine empower staff to deliver high-quality care, especially in resource-limited settings, thus reducing the isolation felt by healthcare workers in remote locations. Medication management systems and electronic health records minimize errors, which enhances staff members’ confidence in their work and satisfaction with patient outcomes. However, social media and messaging tools can also have the opposite effect and potentially reduce job satisfaction and morale.

#### User experience and convenience of using the digital system

User-friendly and seamlessly integrated technologies are preferred, as they reduce learning effort and enhance efficiency. Tools that are mobile-friendly and adaptable to varied workflows improve flexibility and engagement. Conversely, poorly designed and complex interfaces that fail to meet practical needs coupled with inadequate training often decelerate adoption, causing confusion, inefficiencies and errors. A lack of ongoing support frustrates staff, leading to inefficiencies and under-utilization.

## Discussion

This scoping review highlights the complex and multifaceted role of digital technologies in facilitating interprofessional interaction in and among different healthcare settings and on different societal levels. The review aimed to identify, categorize and map the most commonly used technologies for interprofessional interaction and their contextual placement within the adopter-system, considering the potential benefit or value proposition for healthcare professionals. Our findings identified seven primary technology categories for interprofessional interaction. Most implementations occurred at organizational level with fewer at individual, national or international level. The use of digital tools was most frequently reported in oncology-related care settings. Assessing the value of the implemented technologies as well as the impact for the adopter system provided quite a broad spectrum of themes influencing healthcare professionals and workflows.

Few studies provide a comprehensive analysis of interprofessional interaction technologies (4). These technologies present both benefits and challenges, as they can enhance certain aspects of communication while simultaneously introducing new complexities. Across all technologies, enhancing professional interactions emerged as a central theme. Health information technologies were frequently associated with improved health outcomes and continuity of care; however, concerns regarding privacy and confidentiality were also noted (24, 25).

Overall, the included studies mainly paint a very positive picture on the implementation of digital healthcare technologies. As in other research areas, however, many unsuccessful implementations remain unreported or unpublished, leading to a substantial publication bias in this area. Often, new technologies are meant to be a “solution” for an existing problem; as such, many studies focus primarily on solving a specific problem with a certain technology without assessing other aspects such as the usability for end users, sustainable integration, or other potential social consequences. General limitations found in the included studies were small sample sizes, single-institution studies, and low response rates [e.g., (26–30)]. Furthermore, some studies have poor methodological quality and do not use standard measurements for their reported outcome. This does not allow comparisons between studies and also limits reproducibility (26, 31).

One of the primary goals of digitalization in healthcare is to improve collaboration among professionals. While our findings suggest that digital tools contribute positively to teamwork, they do not eliminate all barriers. Successful interprofessional collaboration and interaction cannot be achieved through technology alone. Clear rules and guidelines must be established to facilitate effective teamwork. Furthermore, the consideration of social factors—especially the entanglement between social and technical processes—is essential for developing and implementing information and communication technologies, and to prevent many technology projects from failing to achieve the desired results (32).

At this point, it seems sensible to involve potential users at an early stage in the development and implementation phase. Participatory, human- or user-centered design, or different co-practices (co-design, co-production, co-creation) could be helpful in addressing the social component of development and implementation projects and are increasingly recommended (33, 34). Involving the healthcare professionals as end users early on in the design and implementation process can help ensure that technologies/innovations support rather than hinder their work and are actually used (35, 36). Healthcare technologies should be designed to enhance work processes, minimize administrative burden, and adapt to the complexities of clinical practice. By addressing these challenges, healthcare institutions can foster adoption and ensure that digital transformation in healthcare leads to meaningful improvements in patient care and professional practice.

A positive example of a successful implementation is the Champlain BASE eConsult System (25). The adoption was supported by multiple funding sources, including governmental and institutional funding. The system was implemented gradually, starting with a proof of concept and expanding to full-scale implementation across the health region. Feedback from primary care providers and specialists during early phases informed its development. The service has become integral to reducing wait times for specialist consultations, with primary care providers widely reporting satisfaction with the system. Despite its success, the system relies on ongoing external funding to ensure sustainability (37). This highlights the importance of including the care providers in the development and integration process, as well as the importance of greater institutional and financial support.

Beyond technical implementation, the adoption of digital health tools is deeply influenced by ethical, legal, and socio-cultural factors. The way technologies are designed and introduced can reshape power dynamics among healthcare professionals. While some technologies, such as shared EHRs, can democratize access to information and facilitate collaboration, others may reinforce existing hierarchies by limiting access to certain roles (38). Programmes such as ECHO (Extension for Community Health Outcomes) support medical consultations, education, and clinician collaboration, thus improving patient outcomes and access to care. However, one major concern on the part of physicians participating in an ECHO Project in rural US was that these physicians were intimidated by the number of experts attending the specialist consultation. They were concerned that they would reveal their lack of expertise in public (24).

On the adopter side, most technologies were linked to improved professional interaction and workflow efficiency. eConsults and EHRs were specifically noted for facilitating access to specialist expertise. However, EHRs also posed challenges, including workflow disruptions and usability issues. These findings align with previous research (39), which highlights both the benefits and limitations of EHR access. While improved quality of information was cited as a success factor, studies also reported poor information quality due to inconsistent data models and technical limitations, such as slow loading times and synchronization issues (39). For the purpose of this review, we focused solely on interprofessional interaction between healthcare professionals as the adopter system. However, adopters of healthcare technologies are more than just the healthcare professionals. Implementing technologies in a healthcare system always has indirect effects on patients and caregivers as well. The adoption of digital interaction tools can also enhance patient-provider relationships and foster patient engagement through collaborative, participatory healthcare experiences (40, 41). Digital tools enable staff-efficient care delivery, reduce informal caregiver burden, and address patient isolation, thereby improving the efficiency and flow of patient- centered care. Enhanced communication and transparency build trust and foster empathetic connection, which are critical for effective care. For instance, digital platforms improve access to specialist advice, thus enhancing patient satisfaction and quality of care. Moreover, digital tools empower patients and their families by providing educational resources on health conditions and self-management, thereby fostering patient engagement and enhancing collaboration in the delivery of care. As such, the interaction that occurs via healthcare technology and digital tools between healthcare providers and recipients of healthcare services, their relatives, and associated persons, can also be seen as transdisciplinary interaction. Furthermore, concerns regarding data privacy, compliance with legal frameworks, and the ethical implications of digital interactions should be addressed (42). Messaging tools such as WhatsApp, while convenient, raise significant data security concerns. A regulatory framework that balances accessibility with security is essential in order to ensure trust and compliance among healthcare professionals.

To provide a structured interpretation of these findings, we applied the NASSS framework as an analysis frame (12). In our adaptation, we shifted the focus of the “Condition” domain from disease-specific factors to healthcare settings in order to enable, enabling analysis of how technologies are implemented across different care contexts. Similarly, the “Value Proposition” and “Adopter System” domains were expanded to explicitly address usability, workflow impact, and interprofessional interaction, aligning the framework with our review focus. The findings highlight that digitally supported interprofessional interaction requires multi-level strategies for implementation. The findings indicate that digital technologies can significantly enhance interprofessional interaction, workflow efficiency, and patient- centered care, but also that success depends on alignment with healthcare settings, technology usability, value realization, and workforce support. Policy and organizational strategies should address all four NASSS domains in order to optimize adoption, safety, efficiency, and equity across healthcare systems.

## Limitations

This study has several limitations. It does not assess the direct impact of digital health technologies on patient outcomes, nor does it evaluate their long-term sustainability. However, as a scoping review, our aim was to provide a broad overview of existing literature rather than an in-depth analysis of specific interventions. Furthermore, there is a potential publication bias, as successful implementations are more frequently reported than failures, which may result in an incomplete picture of the challenges associated with digital health adoption. Including unpublished reports or grey literature might have had added benefit in addressing the issue of overly positive reported outcomes. Future studies should focus on longitudinal evaluations to assess the real-world impact and sustainability of healthcare IT systems.

## Conclusion

Digital tools are important for enhancing interprofessional interaction and efficiency in delivering healthcare. However, successful adoption depends on aligning technological design with clinical workflows, involving end-users in development, and addressing regulatory, ethical, and organizational challenges. Some of the studies identified within this scoping review already incorporated elements of participatory technology development, such as involving primary care providers and specialists in the early stages. To further support the successful implementation of healthcare technologies, it is essential to adopt participatory and co-creative approaches that actively integrate user perspectives. These approaches should extend beyond development and pilot testing to also encompass implementation and evaluation phases. This, alongside greater financial and institutional support, might ensure more sustainable and long-term integration of digital health solutions. Furthermore, reflection upon sociocultural differences in the adoption of technology—beyond regulatory and ethical issues—might be beneficial to fully understanding adoption or non-adoption of digital interprofessional collaboration technologies. Particularly sociocultural differences between high-, middle- and low-income countries might provide additional insights into technology development and integration processes.

## Data Availability

The original contributions presented in the study are included in the article/Supplementary Material, further inquiries can be directed to the corresponding author.

## Supplementary material

Supplementary Table 1 Search strategy in detail for PubMed search.

Supplementary Data Sheet 1 Detailed list of all included papers.
