# Supplementary material for: Digitally supported interprofessional interaction in healthcare—a scoping review
Source: Front Digit Health. 2025 Dec 12;7:1688989. doi: 10.3389/fdgth.2025.1688989 (PMC12740914; doi:10.3389/fdgth.2025.1688989)
Supplement: Supplementary Table 1 — Search strategy in detail for PubMed search. [file Table1.pdf]

Supplementary Material

Supplementary Table 1: Search strategy in detail for PubMed search

Participants, concept, context (PCC scheme)	#	Connector	Search String	Hits (11.12.2022)
**Communication and collaboration between different healthcare provider groups**	1		trans-disciplin*[Title/Abstract]	297
	2		transdisciplin*[Title/Abstract]	2,999
	3		cross-disciplinar*[Title/Abstract]	2,216
	4		crossdisciplinar*[Title/Abstract]	2,119
	5		inter-disciplin*[Title/Abstract]	920
	6		interdisciplin*[Title/Abstract]	47,892
	7		multi-disciplin*[Title/Abstract]	8,892
	8		multidisciplin*[Title/Abstract]	119,632
	9		multi-profession*[Title/Abstract]	1,588
	10		multiprofession*[Title/Abstract]	3,234
	11		inter-profession*[Title/Abstract]	1,879
	12		interprofession*[Title/Abstract]	16,172
	13	#1-#12 with OR	(((((((((((trans-disciplin*[Title/Abstract]) OR (transdisciplin*[Title/Abstract])) OR (cross-disciplinar*[Title/Abstract])) OR (crossdisciplinar*[Title/Abstract])) OR (inter-disciplin*[Title/Abstract])) OR (interdisciplin*[Title/Abstract])) OR (multi-disciplin*[Title/Abstract])) OR (multidisciplin*[Title/Abstract])) OR (multi-profession*[Title/Abstract])) OR (multiprofession*[Title/Abstract])) OR (inter-profession*[Title/Abstract])) OR (interprofession*[Title/Abstract])	185,543
	14		"knowledge transfer"[Title/Abstract]	2,591
	15		information*[Title/Abstract]	1,489,287
	16		Health Information Exchange[MeSH Terms]	1,080
	17		cooperat*[Title/Abstract]	171,983
	18		co-operat*[Title/Abstract]	12,063
	19		collaborat*[Title/Abstract]	188,735
	20		communicat*[Title/Abstract]	378,139

	21	#14-#20 with OR	(((((("knowledge transfer"[Title/Abstract]) OR (information*[Title/Abstract])) OR (Health Information Exchange[MeSH Terms])) OR (cooperat*[Title/Abstract])) OR (co-operat*[Title/Abstract])) OR (collaborat*[Title/Abstract])) OR (communicat*[Title/Abstract])	2,089,524
	22	#13 AND #21	(((((((((((trans-disciplin*[Title/Abstract]) OR (transdisciplin*[Title/Abstract])) OR (cross-disciplinar*[Title/Abstract])) OR (crossdisciplinar*[Title/Abstract])) OR (inter-disciplin*[Title/Abstract])) OR (interdisciplin*[Title/Abstract])) OR (multi-disciplin*[Title/Abstract])) OR (multidisciplin*[Title/Abstract])) OR (multi-profession*[Title/Abstract])) OR (multiprofession*[Title/Abstract])) OR (inter-profession*[Title/Abstract])) OR (interprofession*[Title/Abstract])) AND ((((((("knowledge transfer"[Title/Abstract]) OR (information*[Title/Abstract])) OR (Health Information Exchange[MeSH Terms])) OR (cooperat*[Title/Abstract])) OR (co-operat*[Title/Abstract])) OR (collaborat*[Title/Abstract])) OR (communicat*[Title/Abstract]))	52,850
	23		"integrated care"[Title/Abstract]	6,670
	24		Intersectoral Collaboration[MeSH Terms]	2,567
	25		Interdisciplinary Communication[MeSH Terms]	18,111
	26	#23 OR #24 OR #25	(("integrated care"[Title/Abstract]) OR (Intersectoral Collaboration[MeSH Terms])) OR (Interdisciplinary Communication[MeSH Terms])	26,343
	27	#22 OR #26	((((((((((((trans-disciplin*[Title/Abstract]) OR (transdisciplin*[Title/Abstract])) OR (cross-disciplinar*[Title/Abstract])) OR (crossdisciplinar*[Title/Abstract])) OR (inter-disciplin*[Title/Abstract])) OR (interdisciplin*[Title/Abstract])) OR (multi-disciplin*[Title/Abstract])) OR (multidisciplin*[Title/Abstract])) OR (multi-profession*[Title/Abstract])) OR (multiprofession*[Title/Abstract])) OR (inter-profession*[Title/Abstract])) OR (interprofession*[Title/Abstract])) AND ((((((("knowledge transfer"[Title/Abstract]) OR (information*[Title/Abstract])) OR (Health Information Exchange[MeSH Terms])) OR (cooperat*[Title/Abstract]))	75,493

			OR (co-operat*[Title/Abstract])) OR (collaborat*[Title/Abstract])) OR (communicat*[Title/Abstract]))) OR ((("integrated care"[Title/Abstract]) OR (Intersectoral Collaboration[MeSH Terms])) OR (Interdisciplinary Communication[MeSH Terms]))	
Digital tools	28		Health Information Systems[MeSH Terms]	1,587
	29		Ambulatory Care Information Systems[MeSH Terms]	1,171
	30		Information Technology[MeSH Terms]	733
	31		technolog*[Title/Abstract]	626,873
	32		socio-techni*[Title/Abstract]	745
	33		sociotechni*[Title/Abstract]	1,547
	34		mHealth[Title/Abstract]	8,867
	35		eHealth[Title/Abstract]	8,982
	36		digit*[Title/Abstract]	252,283
	37		Electronic Health Records[MeSH Terms]	26,794
	38		Public Health Informatics[MeSH Terms]	1,250
	39		messag*[Title/Abstract]	71,943
	40		messeng*[Title/Abstract]	82,691
	41		app[Title/Abstract]	37,310
	42		video*[Title/Abstract]	160,804
	43		phone[Title/Abstract]	32,395
	44		E-Mail*[Title/Abstract]	9,648
	45		"E Mail"[Title/Abstract]	7,910
	46		"E Mails"[Title/Abstract]	895
	47		Email*[Title/Abstract]	21,852
	48		"electronic mail"[Title/Abstract]	823
	49		"electronic mails"[Title/Abstract]	16
	50		"social media"[Title/Abstract]	25,573
	51		WhatsApp[Title/Abstract]	1,436
	52		Facebook[Title/Abstract]	6,031
	53		Viber[Title/Abstract]	31
	54		WeChat[Title/Abstract]	948
	55		Telegram[Title/Abstract]	168
	56		Kakaotalk[Title/Abstract]	5
	57	#28-#56 with OR	((((((((((((((((((((((((((Health Information Systems[MeSH Terms]) OR (Ambulatory Care Information Systems[MeSH Terms])) OR (Information Technology[MeSH Terms])) OR (technolog*[Title/Abstract])) OR (socio-techni*[Title/Abstract])) OR (sociotechni*[Title/Abstract])) OR (mHealth[Title/Abstract])) OR (eHealth[Title/Abstract])) OR (digit*[Title/Abstract])) OR (Electronic	1,254,069

			Health Records[MeSH Terms])) OR (Public Health Informatics[MeSH Terms])) OR (messag*[Title/Abstract])) OR (messeng*[Title/Abstract])) OR (app[Title/Abstract])) OR (video*[Title/Abstract])) OR (phone[Title/Abstract])) OR (E-Mail*[Title/Abstract])) OR (Email*[Title/Abstract])) OR ("E Mail"[Title/Abstract])) OR ("E Mails"[Title/Abstract])) OR ("Electronic Mail"[Title/Abstract])) OR ("Electronic Mails"[Title/Abstract])) OR ("social media"[Title/Abstract])) OR (WhatsApp[Title/Abstract])) OR (Facebook[Title/Abstract])) OR (Viber[Title/Abstract])) OR (WeChat[Title/Abstract])) OR (Telegram[Title/Abstract])) OR (Kakaotalk[Title/Abstract])	
Healthcare setting	58		health*[Title/Abstract]	3,478,328
	59		hospital*[Title/Abstract]	1,566,327
	60		care*[Title/Abstract]	2,049,851
	61		caring[Title/Abstract]	49,262
	62	#58-#61 with OR	(((health*[Title/Abstract]) OR (hospital*[Title/Abstract])) OR (care*[Title/Abstract])) OR (caring[Title/Abstract])	5,596,504
Total	63	#27 AND #57 AND #62	((((((((((((((trans-disciplin*[Title/Abstract]) OR (transdisciplin*[Title/Abstract])) OR (cross-disciplinar*[Title/Abstract])) OR (crossdisciplinar*[Title/Abstract])) OR (inter-disciplin*[Title/Abstract])) OR (interdisciplin*[Title/Abstract])) OR (multi-disciplin*[Title/Abstract])) OR (multidisciplin*[Title/Abstract])) OR (multi-profession*[Title/Abstract])) OR (multiprofession*[Title/Abstract])) OR (inter-profession*[Title/Abstract])) OR (interprofession*[Title/Abstract])) AND ((((((("knowledge transfer"[Title/Abstract]) OR (information*[Title/Abstract])) OR (Health Information Exchange[MeSH Terms])) OR (cooperat*[Title/Abstract])) OR (co-operat*[Title/Abstract])) OR (collaborat*[Title/Abstract])) OR (communicat*[Title/Abstract]))) OR ((("integrated care"[Title/Abstract]) OR (Intersectoral Collaboration[MeSH	7,261

			Terms])) OR (Interdisciplinary Communication[MeSH Terms]))) AND (((((((((((((((((((((((((((((Health Information Systems[MeSH Terms]) OR (Ambulatory Care Information Systems[MeSH Terms])) OR (Information Technology[MeSH Terms])) OR (technolog*[Title/Abstract])) OR (socio-techni*[Title/Abstract])) OR (sociotechni*[Title/Abstract])) OR (mHealth[Title/Abstract])) OR (eHealth[Title/Abstract])) OR (digit*[Title/Abstract])) OR (Electronic Health Records[MeSH Terms])) OR (Public Health Informatics[MeSH Terms])) OR (messag*[Title/Abstract])) OR (messeng*[Title/Abstract])) OR (app[Title/Abstract])) OR (video*[Title/Abstract])) OR (phone[Title/Abstract])) OR (E-Mail*[Title/Abstract])) OR ("E Mail"[Title/Abstract])) OR ("E Mails"[Title/Abstract])) OR (Email*[Title/Abstract])) OR ("electronic mail"[Title/Abstract])) OR ("electronic mails"[Title/Abstract])) OR ("social media"[Title/Abstract])) OR (WhatsApp[Title/Abstract])) OR (Facebook[Title/Abstract])) OR (Viber[Title/Abstract])) OR (WeChat[Title/Abstract])) OR (Telegram[Title/Abstract])) OR (Kakaotalk[Title/Abstract]))) AND ((((health*[Title/Abstract]) OR (hospital*[Title/Abstract])) OR (care*[Title/Abstract])) OR (caring[Title/Abstract]))	
	64		Filters: from 2012 onwards, English, German, French, Portuguese, Spanish	5,694

Table 2: Search strategy for individual databases

Database	Search String	Filters used	Hits with filters
**CINAHL**	(((MH (intersectoral collaboration) OR TI ("integrated care" OR trans-disciplin* OR transdisciplin* OR cross-disciplinar* OR crossdisciplinar* OR inter-disciplin* OR interdisciplin* OR multi-disciplin* OR multidisciplin* OR multiprofession* OR multi-profession* OR inter-profession* OR interprofession*) OR AB ("integrated care" OR trans-disciplin* OR transdisciplin* OR cross-disciplinar* OR crossdisciplinar* OR inter-disciplin* OR interdisciplin* OR multi-disciplin* OR multidisciplin* OR multiprofession* OR multi-profession* OR inter-profession* OR interprofession*))) AND (((MH (health information exchange) OR TI ("knowledge transfer" OR information* OR cooperat* OR co-operat* OR collaborat* OR communicat*) OR AB ("knowledge transfer" OR information* OR cooperat* OR co-operat* OR collaborat* OR communicat*))) AND (((MH (health information systems OR ambulatory care information systems OR information technology OR electronic health records OR public health informatics) OR TI (technolog* OR socio-techni* OR sociotechni* OR mHealth OR eHealth OR digit* OR messag* OR messeng* OR app OR video* OR phone OR email OR emails OR "electronic mail" OR "electronic mails" OR "social media" OR whatsapp OR facebook OR viber OR wechat OR OR telegram OR Kakaotalk) OR AB (technolog* OR socio-techni* OR sociotechni* OR mHealth OR eHealth OR digit* OR messag* OR messeng* OR app OR video* OR phone OR email OR emails OR "electronic mail" OR "electronic mails" OR "social media" OR whatsapp OR facebook OR viber OR wechat OR OR telegram OR Kakaotalk))) AND (TI (health OR hospital* OR care* OR caring) OR AB (health OR hospital* OR care* OR caring))	Language: English, German, French, Portuguese, Spanish<br><br>Publication year 2012-2023	2,676
**Embase**	((((('integrated care':ti,ab) OR ('Intersectoral Collaboration':ti,ab)) OR ('Interdisciplinary Communication':ti,ab)) OR ((((((((((((trans-disciplin*:ti,ab) OR (transdisciplin*:ti,ab)) OR (cross-disciplinar*:ti,ab)) OR (crossdisciplinar*:ti,ab)) OR (inter-disciplin*:ti,ab)) OR (interdisciplin*:ti,ab)) OR (multi-disciplin*:ti,ab)) OR (multidisciplin*:ti,ab)) OR (multi-profession*:ti,ab)) OR (multiprofession*:ti,ab)) OR (inter-profession*:ti,ab)) OR (interprofession*:ti,ab)) AND (((((('knowledge transfer':ti,ab) OR (information*:ti,ab)) OR ('Health Information Exchange':ti,ab)) OR (cooperat*:ti,ab)) OR (co-operat*:ti,ab)) OR (collaborat*:ti,ab)) OR	Language: English, German, French, Portuguese, Spanish<br><br>Publication year 2012-2023	8,733

	(communicat*:ti,ab)))) AND (((((((((((((((((((((((((('Health Information Systems':ti,ab) OR ('Ambulatory Care Information Systems':ti,ab)) OR ('Information Technology':ti,ab)) OR (technolog*:ti,ab)) OR (socio-techni*:ti,ab)) OR (sociotechni*:ti,ab)) OR (mHealth:ti,ab)) OR (eHealth:ti,ab)) OR (digit*:ti,ab)) OR ('Electronic Health Records':ti,ab)) OR ('Public Health Informatics':ti,ab)) OR (messag*:ti,ab)) OR (messeng*:ti,ab)) OR (app:ti,ab)) OR (video*:ti,ab)) OR (phone:ti,ab)) OR (E-Mail*:ti,ab)) OR (Email*:ti,ab)) OR ('E Mail':ti,ab)) OR ('E Mails':ti,ab)) OR ('Electronic Mail':ti,ab)) OR ('Electronic Mails':ti,ab)) OR ('social media':ti,ab)) OR (WhatsApp:ti,ab)) OR (Facebook:ti,ab)) OR (Viber:ti,ab)) OR (WeChat:ti,ab)) OR (Telegram:ti,ab)) OR (Kakaotalk:ti,ab))) AND ((((health*:ti,ab) OR (hospital*:ti,ab)) OR (care*:ti,ab)) OR (caring:ti,ab))		
**PubMed**	(((((((((((((trans-disciplin*[Title/Abstract]) OR (transdisciplin*[Title/Abstract])) OR (cross-disciplinar*[Title/Abstract])) OR (crossdisciplinar*[Title/Abstract])) OR (inter-disciplin*[Title/Abstract])) OR (interdisciplin*[Title/Abstract])) OR (multi-disciplin*[Title/Abstract])) OR (multidisciplin*[Title/Abstract])) OR (multi-profession*[Title/Abstract])) OR (multiprofession*[Title/Abstract])) OR (inter-profession*[Title/Abstract])) OR (interprofession*[Title/Abstract])) AND (((((("knowledge transfer"[Title/Abstract]) OR (information*[Title/Abstract])) OR (Health Information Exchange[MeSH Terms])) OR (cooperat*[Title/Abstract])) OR (co-operat*[Title/Abstract])) OR (collaborat*[Title/Abstract])) OR (communicat*[Title/Abstract]))) OR ((("integrated care"[Title/Abstract]) OR (Intersectoral Collaboration[MeSH Terms])) OR (Interdisciplinary Communication[MeSH Terms]))) AND (((((((((((((((((((((((((((Health Information Systems[MeSH Terms]) OR (Ambulatory Care Information Systems[MeSH Terms])) OR (Information Technology[MeSH Terms])) OR (technolog*[Title/Abstract])) OR (socio-techni*[Title/Abstract])) OR (sociotechni*[Title/Abstract])) OR (mHealth[Title/Abstract])) OR	Language: English, German, French, Portuguese, Spanish<br><br>Publication year 2012-2023	5,694

	(eHealth[Title/Abstract])) OR (digit*[Title/Abstract])) OR (Electronic Health Records[MeSH Terms])) OR (Public Health Informatics[MeSH Terms])) OR (messag*[Title/Abstract])) OR (messeng*[Title/Abstract])) OR (app[Title/Abstract])) OR (video*[Title/Abstract])) OR (phone[Title/Abstract])) OR (E-Mail*[Title/Abstract])) OR ("E Mail"[Title/Abstract])) OR ("E Mails"[Title/Abstract])) OR (Email*[Title/Abstract])) OR ("electronic mail"[Title/Abstract])) OR ("electronic mails"[Title/Abstract])) OR ("social media"[Title/Abstract])) OR (WhatsApp[Title/Abstract])) OR (Facebook[Title/Abstract])) OR (Viber[Title/Abstract])) OR (WeChat[Title/Abstract])) OR (Telegram[Title/Abstract])) OR (Kakaotalk[Title/Abstract]))) AND ((((health*[Title/Abstract]) OR (hospital*[Title/Abstract])) OR (care*[Title/Abstract])) OR (caring[Title/Abstract]))		
**PsycInfo**	((Title:("integrated care") OR Title:("intersectoral collaboration") OR Title:("interdisciplinary communication") OR Title:(trans-disciplin*) OR Title:(transdiciplin*) OR Title:(cross-diciplinar*) OR Title:(crossdisciplinar*) OR Title:(inter-discipplin*) OR Title:(interdisciplin*) OR Title:(multi-disciplin*) OR Title:(multidisciplin*) OR Title:(multi-profession*) OR Title:(multiprofession*) OR Title:(inter-profession) OR Title:(interprofession)) OR (Abstract:("integrated care") OR Abstract:("intersectoral collaboration") OR Abstract:("interdisciplinary communication") OR Abstract:(trans-disciplin*) OR Abstract:(transdiciplin*) OR Abstract:(cross-diciplinar*) OR Abstract:(crossdisciplinar*) OR Abstract:(inter-discipplin*) OR Abstract:(interdisciplin*) OR Abstract:(multi-disciplin*) OR Abstract:(multidisciplin*) OR Abstract:(multi-profession*) OR Abstract:(multiprofession*) OR Abstract:(inter-profession) OR Abstract:(interprofession))) AND ((Title:(health*) OR Title:(hospital*) OR Title:(care*) OR Title:(caring)) OR (Abstract:(health*) OR Abstract:(hospital*) OR Abstract:(care*) OR Abstract:(caring))) AND ((Title:("Health Information Systems") OR Title:("ambulatory care information systems") OR Title:(technolog*) OR Title:(socio-techni*) OR Title:(sociotechni*) OR Title:(mHealth) OR Title:(eHealth) OR Title:(digit*) OR Title:("electronic health records") OR Title:("public health informatics") OR Title:(messag*) OR Title:(messeng*) OR Title:(app) OR Title:(video*) OR Title:(phone) OR Title:(e-mail) OR Title:(email) OR Title:(e-mails) OR Title:(emails) OR	Language: English, German, French, Portuguese, Spanish<br><br>Publication year 2012-2023	1,617

	Title:("electronic mail") OR Title:("electronic mails") OR Title:("social media") OR Title:(whatsapp) OR Title:(facebook) OR Title:(Viber) OR Title:(wechat) OR Title:(Telegram) OR Title:(Kakaotalk)) OR (Abstract:("Health Information Systems") OR Abstract:("ambulatory care information systems") OR Abstract:(technolog*) OR Abstract:(socio-techni*) OR Abstract:(sociotechni*) OR Abstract:(mHealth) OR Abstract:(eHealth) OR Abstract:(digit*) OR Abstract:("electronic health records") OR Abstract:("public health informatics") OR Abstract:(messag*) OR Abstract:(messeng*) OR Abstract:(app) OR Abstract:(video*) OR Abstract:(phone) OR Abstract:(e-mail) OR Abstract:(email) OR Abstract:(e-mails) OR Abstract:(emails) OR Abstract:("electronic mail") OR Abstract:("electronic mails") OR Abstract:("social media") OR Abstract:(whatsapp) OR Abstract:(facebook) OR Abstract:(Viber) OR Abstract:(wechat) OR Abstract:(Telegram) OR Abstract:(Kakaotalk)))		
**SCOPUS**	(((((TITLE-ABS("integrated care")) OR (TITLE-ABS-KEY("Intersectoral Collaboration"))) OR (TITLE-ABS-KEY("Interdisciplinary Communication"))) OR ((((((((((((TITLE-ABS(trans-disciplin*)) OR (TITLE-ABS(transdisciplin*))) OR (TITLE-ABS(cross-disciplinar*))) OR (TITLE-ABS(crossdisciplinar*))) OR (TITLE-ABS(inter-disciplin*))) OR (TITLE-ABS(interdisciplin*))) OR (TITLE-ABS(multi-disciplin*))) OR (TITLE-ABS(multidisciplin*))) OR (TITLE-ABS(multi-profession*))) OR (TITLE-ABS(multiprofession*))) OR (TITLE-ABS(inter-profession*))) OR (TITLE-ABS(interprofession*))) AND (((((((TITLE-ABS("knowledge transfer")) OR (TITLE-ABS(information*))) OR (TITLE-ABS-KEY("Health Information Exchange"))) OR (TITLE-ABS(cooperat*))) OR (TITLE-ABS(co-operat*))) OR (TITLE-ABS(collaborat*))) OR (TITLE-ABS(communicat*))))) AND ((((((((((((((((((((((((((((TITLE-ABS-KEY("Health Information Systems")) OR (TITLE-ABS-KEY("Ambulatory Care Information Systems"))) OR (TITLE-ABS-KEY("Information Technology"))) OR (TITLE-ABS(technolog*))) OR (TITLE-ABS(socio-techni*))) OR (TITLE-ABS(sociotechni*))) OR (TITLE-ABS(mHealth))) OR (TITLE-ABS(eHealth))) OR (TITLE-ABS(digit*))) OR (TITLE-ABS-KEY("Electronic Health Records"))) OR (TITLE-ABS-KEY("Public Health Informatics"))) OR (TITLE-ABS(messag*))) OR (TITLE-ABS(messeng*))) OR (TITLE-ABS(app))) OR (TITLE-ABS(video*))) OR (TITLE-ABS(phone))) OR (TITLE-ABS(E-Mail*))) OR (TITLE-ABS(Email*))) OR (TITLE-ABS("E Mail"))) OR (TITLE-ABS("E Mails"))) OR (TITLE-ABS("Electronic Mail"))) OR	Language: English, German, French, Portuguese, Spanish<br><br>Publication year 2012-2023	8,090

	(TITLE-ABS("Electronic Mails"))) OR (TITLE-ABS("social media"))) OR (TITLE-ABS(WhatsApp))) OR (TITLE-ABS(Facebook))) OR (TITLE-ABS(Viber))) OR (TITLE-ABS(WeChat))) OR (TITLE-ABS(Telegram))) OR (TITLE-ABS(Kakaotalk)))) AND ((((TITLE-ABS(health*)) OR (TITLE-ABS(hospital*))) OR (TITLE-ABS(care*))) OR (TITLE-ABS(caring)))		
